# LATE-LIFE SOCIAL NETWORKS AND INCIDENT ALZHEIMER'S DISEASE: THE KUAKINI HONOLULU-ASIA AGING STUDY

**DOI:** 10.1093/geroni/igac059.957

**Published:** 2022-12-20

**Authors:** Kalpana Kallianpur, Kamal Masaki, Randi Chen, Bradley Willcox, Richard Allsopp, Philip Davy, Hiroko Dodge

**Affiliations:** Kuakini Medical Center, Honolulu, Hawaii, Honolulu, Hawaii, United States; Kuakini Medical Center, Honolulu, Hawaii, United States; Kuakini Medical Center, Honolulu, Hawaii, United States; Kuakini Medical Center, Honolulu, Hawaii, United States; University of Hawaii, Honolulu, Hawaii, United States; Kuakini Medical Center, Honolulu, Hawaii, United States; Oregon Health & Science University, Portland, Oregon, United States

## Abstract

We assessed longitudinal associations between social networks and incidence of all-cause dementia, Alzheimer’s disease (AD) and vascular dementia in Kuakini Honolulu-Asia Aging Study participants over a 10-year follow-up period. Median split of Lubben Social Network Scale (LSNS) scores defined weak/strong social networks among 2636 men who were dementia-free at baseline (median age 77 years) and during the first 3 years. Kaplan-Meier curves showed that those with strong networks at baseline were less likely to develop all-cause dementia (p<0.0001) and AD (p=0.0006); probability of dementia-free survival at 10 years for strong and weak social network groups was 93.8% and 89.0%, respectively. Cox regression models adjusting for age and other baseline factors revealed associations of weak networks with increased risk of all-cause dementia (HR=1.52, 95%CI=1.11-2.08, p=0.009) and AD (HR=1.67, 95%CI=1.11-2.51, p=0.014). As strong social networks may protect against incident dementia and AD, and are associated with other health benefits, prevention of social isolation of older adults should be considered a priority.

